# Serum levels of tau protein increase according to the severity of the injury in DAI rat model

**DOI:** 10.12688/f1000research.21132.1

**Published:** 2020-01-20

**Authors:** Keisuke Tomita, Taka-aki Nakada, Taku Oshima, Rui Kawaguchi, Shigeto Oda

**Affiliations:** 1 Department of Emergency and Critical Care Medicine, Chiba University Graduate School of Medicine, 1-8-1 Inohana, Chuo, Chiba, 260-8677, Japan

**Keywords:** serum tau protein, diffuse axonal injury, traumatic brain injury, biomarker

## Abstract

Traumatic brain injury (TBI) in the form of diffuse axonal injury (DAI) is difficult to diagnose in the early phase of the injury. Early diagnosis of DAI may provide opportunity for developing treatment and management strategies. Tau protein has been demonstrated to increase in the early phase of TBI with high diagnostic accuracy in patients with DAI. We tested the biological plausibility of tau protein using a rat DAI model by evaluating the association between serum tau levels and the severity of brain injury. DAI was induced in animals using the Marmarou model. After a survival of 60 minutes, rats were anesthetized and sacrificed after obtaining blood samples (5ml) from the heart. Eighteen rats were employed in the present study and were randomly subjected to sham-operated control (n=4), mild DAI (n=7), and severe DAI (n=7). Of seven severe DAI rats, two rats that had focal injury caused by skull fracture were excluded in the measurement of tau protein level. The serum levels of tau protein in the rat DAI model were found to increase significantly and consistently according to the severity of the injury. Rats with DAI showed significantly higher serum levels of tau protein compared to sham rats; the severe DAI rats had higher levels of tau than moderate DAI and sham rats (sham vs. mild, *P*=0.02; mild vs. severe, *P*=0.02). In conclusion, serum tau protein levels may be useful as a biomarker for diagnosing and estimating the severity of DAI in the early phase.

## Introduction

Traumatic brain injury (TBI) in the form of diffuse axonal injury (DAI) is difficult to diagnose with computed tomography (CT) scans, due to the minimal effect on the anatomical structure of the brain in the early phase of the injury. However, DAI is frequently associated with poor clinical outcome, due to the extensive shear disruption of the axons by rotational or acceleration forces of the head ^1, 2^.

Early diagnosis of DAI may contribute to developing treatment and management methods. Apart from imaging diagnostic techniques, biomarkers may be useful in diagnosing DAI and predicting its severity. We focused on tau, a protein which composes important structural elements in the axonal cytoskeleton ^3^. Tau protein has been demonstrated to increase in the early phase of TBI with high diagnostic accuracy in patients with DAI ^4^. In addition, blood tau protein level has been reported to increase in concussion and chronic traumatic encephalopathy ^5, 6^, and it has also been reported that there is an association with severity. However, the same effect has not been confirmed in DAI. Therefore, we tested the biological plausibility of tau protein using a rat DAI model by evaluating the association between serum tau levels and the severity of brain injury.

## Methods

### Ethical statement

The animal study protocol and procedures were reviewed and approved by the Animal Research Committee of Chiba University (approval number 26-341) following the Guidelines for Proper Conduct of Animal Experiments (Science Council of Japan). All procedures used in this study were optimized to minimize animal suffering and distress by carefully following the procedures and monitoring the effectiveness of the analgesics.

### Animals

Male Sprague-Dawley rats (13–14 weeks old, weighing 330–380g) were obtained from CLEA Japan, Inc. A total of eighteen rats were used for this study and were allocated to experimental groups using simple randomization. Each rat was housed one per cage; (270 × 440 × 187 mm; KN-601, Natsume Seisakusyo Co., Ltd., Japan) and standard corn cob cage bedding was used. The rats were kept under 12 h light and dark conditions with water and food *ad libitum* in the animal room at Chiba University, Japan. The diet consisted of standard laboratory feed (CLEA Rodent Diet CA-1, CLEA Japan, Inc.). Temperature (20-24°C) and humidity (50–55%) were also controlled. All rats were checked daily for general physical and health appearance, bedding/water bottle circumstances and any signs of distress.

### DAI model

Each rat was selected randomly from the cage, and the procedures were performed one by one. DAI was induced in animals using the Marmarou model in the laboratory during light conditions ^7^. The Marmarou model is recognized as one of the most commonly used to create DAI in rats. It is inexpensive, easy to perform and capable of producing graded DAI that closely mimics that seen in human TBI. Rats were anesthetized by intraperitoneal injections of medetomidine hydrochloride (0.375mg/kg), midazolam (2mg/kg) and butorphanol tartrate (2.5mg/kg) ^8^. Then, the skull of the rat was exposed with a midline incision to adhere a helmet, a 10 mm diameter stainless steel disk with a thickness of 3mm, at the midline between the coronal and the lambdoid suture. Subsequently, rats were fixed on a foam bed in prone position. A weight (450g) impounder was allowed to fall freely through a Plexiglas guide tube from a predetermined height (severe group 2m; mild group 1m) to provide an impact to the helmet. After the impact, the stainless steel disk was removed and the incision was sutured. Sham-injured rats underwent the same surgical procedure but were not subjected to injury. Rats were housed and warmed at 37.0°C using a heating pad. After a survival time of 60 minutes, DAI and control rats were anesthetized by intraperitoneal injections of medetomidine hydrochloride (0.375mg/kg), midazolam (2mg/kg) and butorphanol tartrate (2.5mg/kg) and sacrificed by the method of cervical dislocation after obtaining blood samples (5ml) by puncturing the heart with a 23G needle. Serum was centrifuged at 1000 rpm for 20 min and stored at -80°C until analysis according to the instructions of enzyme linked-immunosorbent assay (ELISA) kit.

### Measurements

Serum levels of tau protein were measured using a commercially available kit based on the principle of the sandwich ELISA (Cat. no. LS-F23602, LSBio, WA) according to the manufacturer’s instructions. Absorbance readings at wavelength 450nm were performed on the automated plate reader SpectraMax ^®^ M5e with SoftMax Pro Ver. 5.2. Measurements were performed in duplicates.

### Statistical analysis

 In the present study, serum tau protein levels of tau were compared between control and mild injury and between mild and severe injury using Mann-Whitney’s U-test. Since there was no data available to calculate the sample size from previous studies, two cases were first tested in each group. From these results, the estimated level of serum tau protein was 1300±200 pg/mL in the severe DAI group. Furthermore, we also estimated tau protein levels at 900 pg/mL in the mild DAI group and 500 pg/mL in the sham-injured group. In order to achieve the level of statistical significance of 0.05 with a power of 80%, data for four rats in each group were needed. A previous study had reported that death or skull fracture occurred in about 40% of the severe DAI group ^7^. Therefore, we planned to use seven rats for the experiment in the severe DAI group. In addition, seven rats were used in the mild group in case of any adverse events occurring. All statistical analysis was performed with the GraphPad Prism 7 (GraphPad Software, San Diego, CA, USA).

## Results

**Figure 1.**
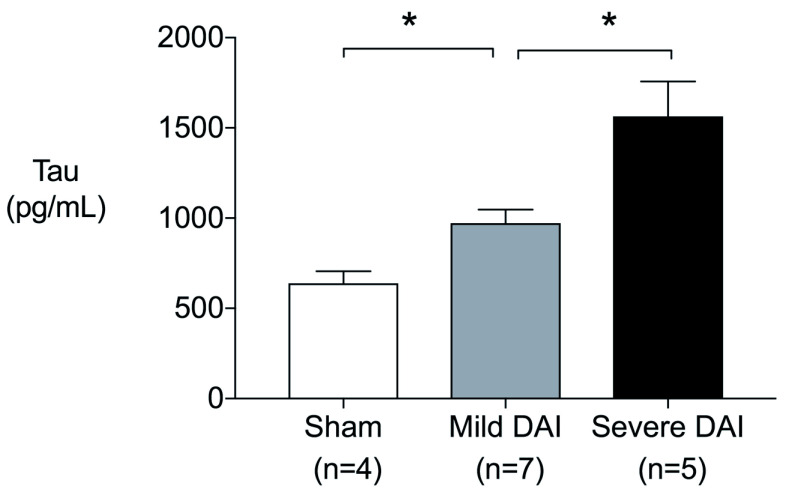
Serum tau protein levels in diffuse axonal injury model rats. Serum tau protein levels increased significantly as degree of diffuse axonal injury increased at one hour after injury (sham vs. mild, *P*=0.024, sham vs. severe, *P*=0.016, mild vs. severe, *P*=0.018). *P* values were calculated using Mann-Whitney’s U-test. Error bars indicate inter-quartile range. * *P*<0.05.

Eighteen rats were employed in the present study and were randomly subjected to sham-operated control (n=4), mild DAI (n=7), and severe DAI (n=7). The mean weight ± standard deviation (SD) and age ± SD in each group was 360.0±8.2g, 365.7±13.9g and 360.6±13.7g, and 94.3±2.6 postnatal days, 93.1±1.1 postnatal days and 93.2±1.3 postnatal days, respectively ^9^. Of seven severe DAI rats, two rats that had focal injury caused by skull fracture were excluded in the measurement of tau protein level. The serum levels of tau protein in the rat DAI model were found to increase significantly and consistently according to the severity of the injury ( Figure 1).

## Discussion

In our study, the serum tau protein levels in the rat DAI model were found to increase significantly and consistently according to the severity of the injury.

The potential biomarkers of DAI such as S-100 calcium-binding protein B, neuron specific enolase, neuron filament, etc., have been evaluated for usefulness for diagnosis and prognostication, with limited success to date ^10– 12^. Tau protein is a microtubule-associated protein and has highly specific expression in neuronal axons ^3^. In a previous animal study, serum tau levels were higher in rats subjected to focal brain injury compared to sham operated controls. The highest level was observed one hour after injury, compared to values at 6h, 24h, 48h, and 168h. The serum tau protein levels increased according to the severity of the injury (sham vs. mild, *P*<0.001; mild vs. severe, *P*<0.001), which was consistent with our results despite the difference in the injury model ^13^. However, to the best of our knowledge, no study has demonstrated the relationship between the severity of DAI and serum tau protein level. By using the rat DAI model rat, we were able to demonstrate that the serum level of tau protein increases significantly and consistently according to the severity of DAI.

There are several limitations in this study. In Marmarou’s study, the severity of traumatic brain injury of rats was differentiated by the height of the weight drop and confirmed according to the mortality after impact. Although we followed the same procedures for producing the models in this study, we did not evaluate the severity of DAI. Furthermore, we only evaluated serum tau protein level at a single time point of one hour from traumatic brain injury. However, setting multiple timepoints may have contributed to a more precise evaluation of the relationship between serum tau protein level and severity of DAI.

In conclusion, the serum levels of tau protein may be a useful biomarker for diagnosing and estimating the severity of DAI in the early phase.

## Data availability

### Underlying data

DRYAD: Serum levels of tau protein increase according to the severity of the injury in DAI rat model. https://doi.org/10.5061/dryad.3r2280gc9 ^9^.

Data are available under the terms of the Creative Commons Zero "No rights reserved" data waiver (CC0 1.0 Public domain dedication).
