# Supplementary material for: Serum levels of tau protein increase according to the severity of the injury in DAI rat model
Source: F1000Res. 2020 Jan 20;9:29. [Version 1] doi: 10.12688/f1000research.21132.1 (PMC7707115; doi:10.12688/f1000research.21132.1)
Supplement: Peer review history

# Peer review: Reviewer response for version 1

**DOI:** 10.5256/f1000research.23261.r74639

**Published:** 2020-11-30

**Authors:** Amanda Heslegrave

**Affiliations:** 1 Department of Neurodegenerative Diseases, University College London, London, UK

This study aims to investigate whether serum tau could be a useful biomarker for estimating the severity of diffuse axonal injury in the early stages after trauma. The study uses a method to induce injury which depends on a weight dropped from a height to provide impact onto a helmet fixed to the rats head; two different heights were used.

 The design of the experiment is simple and also the outcome was one serum tau measurement. The experiment assumes that the rats in each group receive the same injury - which is not unreasonable but is there a way to check this? Also the threshold for injury could have been tested by using more height conditions.

 Overall the conclusions do not seem unreasonable; that tau is increased quickly in serum following neuronal injury.

Is the work clearly and accurately presented and does it cite the current literature?

Yes

If applicable, is the statistical analysis and its interpretation appropriate?

Yes

Are all the source data underlying the results available to ensure full reproducibility?

Yes

Is the study design appropriate and is the work technically sound?

Yes

Are the conclusions drawn adequately supported by the results?

Yes

Are sufficient details of methods and analysis provided to allow replication by others?

Yes

Reviewer Expertise:

Biomarkers of neurodegenerative disease

I confirm that I have read this submission and believe that I have an appropriate level of expertise to confirm that it is of an acceptable scientific standard.

# Peer review: Reviewer response for version 1

**DOI:** 10.5256/f1000research.23261.r74640

**Published:** 2020-11-20

**Authors:** Mieszko Olczak

**Affiliations:** 1 Department of Forensic Medicine, Medical University of Warsaw, Warsaw, Poland

The study evaluates the association between serum tau levels and the severity of brain injury in an animal model.

 Methodology, statistical analysis, results, and discussion are done correctly. One of the drawbacks of the work is the small size of research groups. It should be assumed that the differences between animals within the research groups are minimal and the results are reproducible. I have no objections.

Is the work clearly and accurately presented and does it cite the current literature?

Yes

If applicable, is the statistical analysis and its interpretation appropriate?

Yes

Are all the source data underlying the results available to ensure full reproducibility?

Yes

Is the study design appropriate and is the work technically sound?

Yes

Are the conclusions drawn adequately supported by the results?

Yes

Are sufficient details of methods and analysis provided to allow replication by others?

Yes

Reviewer Expertise:

Central nervous system, forensic medicine, trauma

I confirm that I have read this submission and believe that I have an appropriate level of expertise to confirm that it is of an acceptable scientific standard.

# Peer review: Reviewer response for version 1

**DOI:** 10.5256/f1000research.23261.r69132

**Published:** 2020-08-17

**Authors:** Vassilis E Koliatsos

**Affiliations:** 1 Departments of Pathology, Neurology, and Psychiatry and Behavioral Sciences, Johns Hopkins University, Baltimore, MD, USA

This is a simple straightforward report on the serum levels of tau protein, measured by ELISA, in two different settings of the impact-acceleration model of diffuse traumatic brain injury (presumably causing two degrees of severity of diffuse axonal injury [DAI]). The advantage of having such data available is to show that we may have (emphasis is on *may*) a peripheral reporter of the severity of diffuse TBI and, potentially, DAI, using a model that is very appropriate for the task.

 However, as the authors would be the first to acknowledge, this experiment uses only two degrees of severity of impact acceleration (IA) based on mechanical inputs without testing for severity of traumatic brain injury and certainly not severity of DAI by any of the available applicable measures, also without a time course for the observed changes that is essential for characterizing DAI. Therefore, the neuropathology of DAI has not been tested here and it would appear that the only part of the report where something like this could be mentioned is the Discussion—certainly not the title, where a more neutral term such as “severity of impact acceleration” would be more appropriate.

 Also, with only two degrees of IA tested, the conditional “may” should be used throughout. We need more work before we can look at a definitive study linking peripheral tau protein levels to severity of diffuse traumatic brain injury and quite a bit more if we want to explore peripheral markers of DAI.

Is the work clearly and accurately presented and does it cite the current literature?

Yes

If applicable, is the statistical analysis and its interpretation appropriate?

Partly

Are all the source data underlying the results available to ensure full reproducibility?

Yes

Is the study design appropriate and is the work technically sound?

Yes

Are the conclusions drawn adequately supported by the results?

Partly

Are sufficient details of methods and analysis provided to allow replication by others?

Yes

Reviewer Expertise:

Traumatic brain injury, neurodegenerative disease, neuropathology, axonopathy, axons, Wallerian degeneration, brain-behavior correlations

I confirm that I have read this submission and believe that I have an appropriate level of expertise to confirm that it is of an acceptable scientific standard, however I have significant reservations, as outlined above.
